# Evaluation of Docking Target Functions by the Comprehensive Investigation of Protein-Ligand Energy Minima

**DOI:** 10.1155/2015/126858

**Published:** 2015-11-26

**Authors:** Igor V. Oferkin, Ekaterina V. Katkova, Alexey V. Sulimov, Danil C. Kutov, Sergey I. Sobolev, Vladimir V. Voevodin, Vladimir B. Sulimov

**Affiliations:** ^1^Dimonta, Ltd., Nagornaya Street 15, Building 8, Moscow 117186, Russia; ^2^Research Computer Center, Moscow State University, Leninskie Gory 1, Building 4, Moscow 119992, Russia

## Abstract

The adequate choice of the docking target function impacts the accuracy of the ligand positioning as well as the accuracy of the protein-ligand binding energy calculation. To evaluate a docking target function we compared positions of its minima with the experimentally known pose of the ligand in the protein active site. We evaluated five docking target functions based on either the MMFF94 force field or the PM7 quantum-chemical method with or without implicit solvent models: PCM, COSMO, and SGB. Each function was tested on the same set of 16 protein-ligand complexes. For exhaustive low-energy minima search the novel MPI parallelized docking program FLM and large supercomputer resources were used. Protein-ligand binding energies calculated using low-energy minima were compared with experimental values. It was demonstrated that the docking target function on the base of the MMFF94 force field* in vacuo* can be used for discovery of native or near native ligand positions by finding the low-energy local minima spectrum of the target function. The importance of solute-solvent interaction for the correct ligand positioning is demonstrated. It is shown that docking accuracy can be improved by replacement of the MMFF94 force field by the new semiempirical quantum-chemical PM7 method.

## 1. Introduction

Protein-ligand binding free energy calculation is one of the key problems for molecular modeling in the computer-aided structural based drug design [1–4]. However, the accuracy of such calculations better than 1 kcal/mol has not been reached yet for a randomly selected target protein [1]. Only with such high accuracy of the protein-ligand binding free energy calculations is it possible to perform the rational inhibitor optimization on the basis of computer modeling and to advance from weak inhibitors to lead compounds (hit to lead) followed by the lead optimization to increase the binding affinity and to improve the selectivity of new inhibitors. Though the most accurate calculations of the protein-ligand binding free energy can be done with molecular dynamic (MD) simulations [5], other approaches of the protein-ligand binding energy calculations, especially docking, are also in demand. Docking is the molecular modeling method based on the search of the ligand binding pose in the target protein active site and the subsequent calculation of the score, which allows the protein-ligand binding free energy to be estimated. Although appreciable progress in improving accuracy of protein-ligand binding free energy calculations with docking is visible in recent years, for example, see [6, 7], the success rate, but not high accuracy, is still a measure of the docking success in ligand positioning as well as in the ligand binding energy calculation [7]. It is not surprising because the accuracy of such calculations depends on many interrelated factors in a complicated manner. Those factors are the force field describing inter- and intramolecular interactions, the solvent (water) model, the target protein and ligand models, method and approximations of the free energy calculation, and algorithms of calculations and computing resources concentrated on solving the docking problem for one protein-ligand pair, and so forth.

In the frame of the docking procedure, the ligand binding pose is generally believed to be the global minimum of the protein-ligand potential energy function (the docking paradigm). Thus, the ligand positioning is the global minimum search problem for the energy target function, depending on the degrees of freedom of the given protein-ligand system. Due to thermal motion in the thermodynamic equilibrium state, the ligand continuously jumps from one binding pose to another and for binding energy estimation we have to find not only the energy global minimum but also at least the low-energy part of the whole local minima spectrum.

The target function is defined by the choice of either the force field or the quantum-chemical method describing inter- and intramolecular interactions and also by solvent, target protein, and ligand models. Obviously, high accuracy of the correct ligand positioning is the necessary condition for high accuracy of the protein-ligand binding energy calculation and the latter is vitally important for high reliability of docking programs and high effectiveness of their application in drugs design. So, the adequate choice of the target function is extremely important for high accuracy of docking.

There is a wide variety of docking programs, such as AutoDock [8, 9], FlexX [10], FlexE [11], ICM [12, 13], DOCK [7, 14], GOLD [15], SOL [16–18], TTDock [19], BUDE [20], and Surflex-Dock [21] with their own target functions and the global minimum search algorithms for the ligand positioning. The situation is aggravated by the fact that most of the target functions used in these docking programs in addition to force field parameters have usually some extra parameters fitted for better predictions at a certain training set of proteins and ligands. These fitting parameters have no physical sense, and their usage makes it difficult to estimate* a priori* the ligand positioning accuracy as well as the accuracy of the protein-ligand binding energy calculations for a given force field.

In this work, we evaluated 5 target functions for ligand-protein docking on the base of the MMFF94 force field (Merck molecular force field) [22] in vacuum, on the base of the PM7 quantum-chemical semiempirical method in vacuum [23] and also taking into account several implicit solvent models: PCM [24, 25], COSMO [26], and SGB [27, 28]. These target functions were used without any fitting parameters for the same proteins and ligands structural models. As a global optimization algorithm, we chose the simple Monte Carlo search method to perform the comprehensive search of the protein-ligand low-energy local minima. This search method was implemented in the novel FLM (Find Local Minima) docking program [29]. The FLM program is too slow to compete with existing docking programs but it can find all low-energy local minima in a given part of the protein-ligand phase space if large enough computer resources are available.

The detailed examination of the low-energy minima spectra of a set of protein-ligand complexes has been fulfilled due to use of large supercomputer resources concentrated for this task at the Lomonosov supercomputer of Lomonosov Moscow State University.

## 2. Methods

### 2.1. The FLM Program

The FLM program is the MPI (message passing interface) based program, developed to find low-energy minima of the ligand-protein system. During the minima search, the protein is considered as rigid and the ligand is fully flexible.

#### 2.1.1. Minima Search Algorithm

The FLM program finds local energy minima of the protein-ligand complex by the simple Monte Carlo search algorithm: multiple local optimizations are performed starting from random initial ligand positions. The random initial ligand position is obtained by a random continuous ligand deformation and rotation-translation:

(i) The ligand geometrical center is moved to a random point in the search area. The geometrical center of a molecule is defined here as its center of gravity with all atomic masses equal to unity. The search area is defined as the sphere with the center at the ligand native position geometrical center and with the radius of 8 Å. The ligand native position is the position of the ligand in the crystallized protein-ligand complex structure. The search area sphere covers the active site of the target protein.
(ii) The ligand is rotated as a whole around a random axis passing through the ligand center by a random angle from [−*π*, *π*].
(iii) The ligand torsions are rotated by a random angle from [−*π*, *π*] (torsion is a single acyclic bond of the ligand).

Not all random system conformations are further optimized. At first, atom-atom distances are checked: atoms from each ligand-ligand or protein-ligand atom pair must be separated by more than 0.5 Å. Otherwise, this random system conformation is rejected. Local optimization is performed by the L-BFGS (limited-memory Broyden-Fletcher-Goldfarb-Shanno) [30, 31] algorithm without any restrictions on the positions of the ligand atoms in the search area. All Cartesian coordinates of ligand atoms are moved during optimization. Each local optimization stopped when the maximal component of the optimized target function gradient decreased to the value 10^−5 ^kcal/mol/Å. If the ligand center moves out of the search area after the optimization, the respective local minimum is rejected.

After successful local optimization, the energy minimum can be recalculated taking into account interaction with solvent; see Section 2.2.

A set of computed different local minima with the lowest potential energies is being kept in operative memory during FLM calculations. A new computed local minimum is included into the set, if it differs from any minimum of the set, and the minimum with the highest energy is excluded from this set. Two minima are different if RMSD (root mean square deviation) between them exceeds 0.1 Å. The RMSD is calculated over the ligand heavy atoms without taking into account possible chemical symmetry. The minima search for each protein-ligand complex was conducted during the given time interval: 3 hours. This way of the program halt was used due to some peculiarities of our supercomputer queuing system.

#### 2.1.2. Parallelization Algorithm and Efficiency

The local minima search is parallelized: independent local optimizations of different initial ligand conformations are continuously performed in parallel by different MPI processes. The optimization results are collected in the master process to form the low-energy minima set. The current collected minima set is repeatedly sent back from the master process to other processes, so other processes can select only promising minima to send.

Calculations were done on the “Lomonosov” supercomputer [32]: 1024 nodes (8192 CPUs) were utilized for each run of the FLM program and about 20 000 CPU *∗* hours per a protein-ligand complex and about 100 CPU *∗* hours per a free ligand were consumed during these calculations. Total time for obtaining all minima sets was about several million CPU *∗* hours (including time of postprocessing minima energies recalculations with the other programs: MCBHSOLV and MOPAC). Parallelization efficiency can be estimated via number of finished optimizations in a fixed time.  Table 1 presents amount of “useful” calculations in a fixed time of 3 hours (number of finished local optimizations) depending on number of used nodes for the 1DWC protein-ligand complex.

As we can see in Table 1, the FLM program performance scales linearly with the increasing number of working processes. So, the chosen simple Monte Carlo search method for the global optimization is justified in terms of scalability.

### 2.2. Minima Obtaining Protocol

The set of 16 protein-ligand complexes with experimentally known structures and binding constants was chosen from the Protein Data Bank (PDB) [33] for low-energy local minima search:

- 4 complexes of the CHK1 (checkpoint kinase 1) protein (4FT0, 4FT9, 4FSW, and 4FTA).
- 2 complexes of the ERK2 (extracellular signal-regulated kinase 2) protein (4FV5 and 4FV6).
- 2 complexes of the thrombin protein (1DWC and 1TOM).
- 6 complexes of the urokinase protein (1C5Y, 1F5L, 1O3P, 1SQO, 1VJ9, and 1VJA).
- 2 complexes of the factor Xa protein (2P94 and 3CEN).

These protein-ligand complexes were chosen, because they are available in PDB with good resolution, and the ligands variety covers a wide range from small and rigid ligands (4FSW ligand: 26 atoms including hydrogen atoms, 0 torsions) to big and flexible ones (1VJ9 ligand: 74 atoms including hydrogen atoms, 19 torsions). Also, the locally optimized ligand native position has RMSD from the original native pose less than 1.5 Å for all these 16 complexes, both for the optimization with the MMFF94* in vacuo* target function and for the optimization with the PM7* in vacuo* target function. That means that the locally optimized ligand native position still can represent the native ligand pose.

Protein structures were prepared by elimination of all “HETATM” records (i.e., the records corresponding to atoms, ions, and molecules which are not part of the protein structure) from the PDB files of the complexes, and then hydrogen atoms were added by the original APLITE program [16] to the protein structures. The APLITE program adds hydrogen atoms according to the standard amino acid protonation states at pH = 7. Histidine protonation state is chosen by comparing of electrochemical potentials for hydrogen atom at “HD1” and “HE2” positions. Optimization of hydrogen atoms positions is performed with MMFF94 force field after the hydrogen atoms preplacement. During this optimization, all rotation variants of torsionally moveable hydrogen atoms (e.g., hydroxyl hydrogen atom from tyrosine) are tested. The heavy atoms optimization is not performed.

Ligands were also taken from the PDB files. Hydrogen atoms were added to the ligands by Avogadro program [34]. The heavy atoms optimization is not performed for the initial ligand conformation. The table with 2D ligand structures and information about their charges and numbers of atoms and torsions is presented in the Supplementary Material available online at http://dx.doi.org/10.1155/2015/126858.

The target protein was considered as rigid, and the ligand was considered as totally flexible and moveable in the search area (see Section 2.1.1) around its native position during the minima search.

The first set of the low-energy local minima was obtained by the FLM program with the target function from the MMFF94 force field [22]: the local optimization of the random initial ligand position in the search area and the local minimum selection into the minima set were made on the base of the protein-ligand MMFF94 energy value* in vacuo*. This set is designated as “{1}MMFF94”. Then, these local minima were recalculated for the same protein-ligand geometries with the MMFF94 force field target function taking into account the interaction with water solvent by the MCBHSOLV program [35] in the frame of the PCM implicit model. This low-energy local minima set is designated as “{1}MMFF94 + PCM”. The third local minima set was obtained from the “{1}MMFF94” set by the protein-ligand energy local optimization in the frame of the semiempirical quantum-chemical PM7 method [23] with the help of the MOPAC program [36], and this minima set was designated as “{1}PM7”. Finally, energies of all minima from the “{1}PM7” set were recalculated for the same geometries by the PM7 method with the COSMO implicit water solvent model [26] implemented in the MOPAC program, and this minima set was designated as “{1}PM7 + COSMO”.

Further, the second option of the low-energy minima search was performed by the FLM program for all 16 protein-ligand complexes as follows: the selection of local minima into the low-energy minima set was made on the base of the MMFF94 force field taking into account water in the frame of the PCM implicit solvent model. In other words, the local energy optimization of the initial random ligand position in the search area was carried out in the frame of the MMFF94 force field* in vacuo* as previously, but the selection into the low-energy local minima set was made on the base of the minima energies recalculated in the final optimization point taking into account the protein-ligand complex interaction with water solvent in the frame of the PCM model [35]. This set was designated as “{2}MMFF94 + PCM”. Energies of these minima have been recalculated with the MMFF94 force field* in vacuo* (the minima set “{2}MMFF94”) and also taking into account interaction of protein-ligand complexes with water in the frame of the SGB [27] implicit solvent model (the set “{2}MMFF94 + SGB”) implemented in the DISOLV program [16, 28].

In other words, the 1st group of the minima sets has been obtained on the base of MMFF94* in vacuo* target function local minima search: “{1}MMFF94” and “{1}MMFF94 + PCM”, with one ensemble of configurations, and “{1}PM7” and “{1}PM7 + COSMO”, with another ensemble of configurations. The 2nd group of the minima sets has been obtained on the base of the local minima search with the MMFF94 target function taking into account the PCM solvent model: “{2}MMFF94”, “{2}MMFF94 + PCM”, and “{2}MMFF94 + SGB”, all with the same minima configurations.

Implicit solvent models PCM and SGB are implemented in the DISOLV program, and it has been shown that the sufficiently high accuracy (<1 kcal/mol) of the protein-ligand complex interaction with solvent could be achieved only for a sufficiently small (≤0.1–0.3 Å) step of the triangulation grid on the SES (Solvent Excluded Surface) surface [28]. However, the PCM solving in the DISOLV program is too slow to be used in the docking process. Nevertheless, the MCBHSOLV program [35] has been developed recently on the base of the multicharge method for approximation of large dense matrices [37] generated by triangulation of the molecular SES and by discretization of polarized charges on it. This new implementation of the PCM model is about 300 times faster than the DISOLV program without the accuracy loss. Just the MCBHSOLV program has been used for obtaining the minima sets “{1}MMFF94 + PCM” and “{2}MMFF94 + PCM” with the triangulation grid steps 0.2 Å and 0.3 Å, respectively, and in the latter case all selected minima energies have been recalculated with the triangulation grid step 0.15 Å. The minima set “{2}MMFF94 + SGB” has been obtained with the DISOLV program using the SGB method (the triangulation grid step was also 0.15 Å), which was not so accurate as PCM [28] but it is several times faster than MCBHSOLV [35].

Parameters of the FLM minima search were chosen in such a way that 10^5^–10^6^ test local optimizations were performed for one protein-ligand complex and only 10^3^–10^4^ different minima (without taking into account chemical symmetry) were chosen into the low-energy minima set. Among these low-energy minima up to several dozen ones were in the interval 5*kT* from the lowest energy minimum, for example, 46 minima for the 1VJA complex, and only those gave a significant contribution to the protein-ligand binding free energy.

In addition to the low-energy minima found by the FLM program, we considered also the locally optimized ligand poses obtained with the same target functions local optimization but from the experimentally observed native ligand initial positions. Just these optimized native ligands were compared with the respective low-energy minima found by the FLM program.

### 2.3. Binding Free Energy Calculation

The protein-ligand binding free energy Δ*G*_bind_ is calculated as Δ*G*_bind_ = *G*(PL) − *G*(P) − *G*(L), where *G*(PL), *G*(P), and *G*(L) are free energies of the protein-ligand complex, the free protein, and the free ligand, respectively. Proteins are considered as rigid and free energies of protein-ligand complexes and free ligands are calculated in the multiwell approximation which is similar to the “mining minima” method of Chen et al. [6]. The potential energy of a molecular system is approximated by a set of independent parabolic wells in these methods. The multiwell approximation differs from the “mining minima” method mainly by more uniform and exhaustive low-energy local minima search by the FLM program instead of configuration space exploration along a combination of low-frequency modes as it was made by the “mining minima” method; also we used the Cartesian coordinates instead of the bond-angle-torsion coordinates. The configuration integral of a molecular system *Z* (thus, the free energy *G* = −*kT*ln⁡(*Z*)) is approximated by a sum of contributions from different energy wells *Z* ^*i*^:

(1) $$\begin{array}{c} Z=\sum_{i}Z^{i}=\sum_{i}\exp\left(\;-\frac{E_{0}^{i}}{kT}\;\right)Z_{\nu }^{i}Z_{t}^{i}Z_{r}^{i}, \end{array}$$

where *E*_0_^*i*^ is the potential energy value in the minimum of the *i*th energy well, *Z*_*ν*_^*i*^ corresponds to the vibrational degrees of freedom of the ligand in the *i*th energy well, and *Z*_*t*_^*i*^ and *Z*_*r*_^*i*^ correspond to the translation and rotation of a molecular system as a whole, respectively [38, 39]:

(2) $$\begin{array}{c} Z_{\nu }^{i}=\overset{3n}{\underset{l=1}{\prod }}\frac{e^{-\hbar \omega_{l}^{i}/2kT}}{1-e^{-\hbar \omega_{l}^{i}/kT}}, \end{array}$$

where *ω*_*l*_^*i*^ is *l*th natural frequency of *i*th harmonic oscillator corresponding to the *i*th energy well, *n* is the number of vibrating atoms, *k* is the Boltzmann constant, and *T* is absolute temperature:

(3) $$\begin{array}{c} Z_{t}^{i}=e*\frac{{\left(2\pi MkT\right)}^{3/2}}{h^{3}\rho }, \end{array}$$

where *e* is Euler's constant, *M* is the overall mass of the molecular system, *ρ* is the concentration (the reciprocal volume per one molecule; in the case of standard free energy calculation *ρ* = 1 mol/L = 6.02 *∗* 10^26^ units/m^3^), and *h* is Planck's constant:

(4) $$\begin{array}{c} Z_{r}^{i}=\frac{{\left(8\pi^{2}kT\right)}^{3/2}}{h^{3}}\sqrt{\pi I_{A}I_{B}I_{C}}; \end{array}$$

here *I*_*A*_, *I*_*B*_, and *I*_*C*_ are principal moments of inertia of the molecule.

Natural frequencies for protein-ligand complexes and ligands were calculated through respective Hessian matrices. Proteins were considered as rigid, and no frequencies were calculated for them.

## 3. Results and Discussion

### 3.1. Minima Analysis

In principle, the Monte Carlo search algorithm is able to find the truly global minimum at the expense of huge computational resources. If we want to treat the result of the Monte Carlo search as global minimum, then this minimum, at least, must have energy below or equal to the energy of any arbitrary ligand position. The ligand native position is believed to be the global minimum of the protein-ligand system potential energy. So, the optimized ligand native position can be used as good upper estimate of the global minimum energy. We can check whether the Monte Carlo search algorithm has found the minimum with the energy below or equal to the energy of the optimized ligand native position. We checked this statement for both of the the FLM program run results, that is, for “{1}MMFF94” and “{2}MMFF94 + PCM” minima sets, and this statement is true in all cases except the 1VJA case from the “{2}MMFF94 + PCM” minima set. So, we can adopt that the Monte Carlo search parameters were good enough, and the found minima are the real low-energy minima. The exceptional case of the 1VJA complex, where ligand has 61 atoms and 17 torsions, is a hard system for the global minimum search, and the time of the minima search was not long enough to reveal the global minimum.

**Figure 1:**
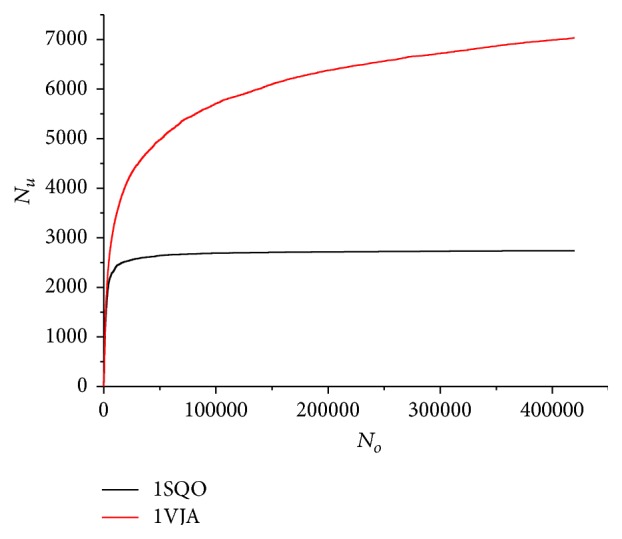
The dependence of the local minima set updates number (*N*_*u*_) on the total number of the performed “test optimizations” (*N*_*o*_) is presented for 1SQO (black lower line) and 1VJA (red higher line) protein-ligand complexes. The saturation means that *N*_*u*_ is not changed with the increase of *N*_*o*_. The target function was the MMFF94 energy in vacuum.

Reliability of the low-energy minima search for a given protein-ligand complex can be verified as follows. Let us analyze the update rate of the local minima set (the set of different local minima with the lowest energies) during “test optimizations.” The local minima set is updated, when a new local minimum after a “test optimization” is accepted into it. If the local minima set is not updated for a long time, then the local minima search is likely thorough. Two examples of the local minima set update rate are shown in Figure 1, where the dependences of the number of updates on the total number of the performed “test optimizations” are presented for two protein-ligand complexes. First example, the protein-ligand complex 1SQO where the ligand has 34 atoms and 4 torsions, is a simple system for the local minima search. Second example, the protein-ligand complex 1VJA where the ligand has 61 atoms and 17 torsions, is a hard system for the local minima search. As we can see from Figure 1, the local minima set for the 1SQO complex almost reached the saturation after 4 *∗* 10^5^ “test optimizations”, but the local minima set for the 1VJA complex was far from the saturation after 4 *∗* 10^5^ “test optimizations.” So, it indicates that the complete minima search for the protein-ligand complex with a big flexible ligand requires more than 4 *∗* 10^5^ “test optimizations.”

It is also interesting to analyze the global minimum update rate. The global minimum of the 1SQO protein-ligand complex was found almost immediately, after 661 done “test optimizations,” and it has not changed till the end. But the global minimum of the 1VJA complex was updated for the last time only after 64205 done “test optimizations.” If we did more than 4 *∗* 10^5^ “test optimizations,” we would probably find a deeper global minimum of the 1VJA complex.

Let us introduce some notations. The minima set of the given protein-ligand complex with energies calculated by a given target function can be sorted by their energy in ascending order; that is, every minimum gets its own index equal to its number in this sorted list of minima. The lowest energy minimum has index equal to 1. When we include the energy of the locally optimized native ligand in this sorted list, it also will get a certain index and we will designate it as “Index of Native” or “IN.” When we do not include the optimized native ligand in this sorted minima list, some minima from the list might be close in space to the native (nonoptimized) ligand position. It is possible even that one minimum found by the FLM program will coincide with the optimized native ligand position. We designate the index of the minimum having RMSD from the nonoptimized native ligand position less than 2 Å as “Index of Near Native” or “INN.” If there are several such minima which are close to the native position, we will choose the minimum with the lowest energy (with the lowest index) as “INN.” The extreme values of these indexes could be interpreted as follows:

- IN = 1 and INN = 1: the target function is valid for ligand positioning, and the minima search is thorough.
- IN = 1 and INN ≫ 1: the minima search is most likely to be incomplete. When the optimized native position has the lowest energy, some near-native positions will certainly have also low energies.
- IN ≫ 1 and INN = 1: there are likely to be experimental inaccuracies in the native ligand position. The target function is most likely valid for ligand positioning, and the minima search is thorough.
- IN ≫ 1 and INN ≫ 1: the target function is invalid for ligand positioning.

The “IN” and “INN” values for all 16 complexes and for 7 target functions are presented in Table 2.

We can see in Table 2 that the protein-ligand MMFF94 energy* in vacuo* is the valid target function for ligand positioning strictly speaking only for few protein-ligand complexes: 1C5Y, 1F5L, and 1SQO (see column “{1}MMFF94”). Only for these three complexes (20% out of 16 complexes; this percentage was the same when we expanded the test set up to 30 complexes) the optimized native ligand position has the lowest energy among all energy minima found by the FLM program (IN = 1), and the position of the minimum with the lowest energy (the global minimum of the target function) found by the FLM program is close to the ligand native pose (INN = 1). For all other 13 complexes, the energy of optimized native ligand position is higher (IN > 1) than energies of some minima of the target function (MMFF94* in vacuo*) and the difference between the optimized native ligand position and the global energy minimum can be several kcal/mol (4.7 kcal/mol for the 1VJA complex) or as large as 91.2 kcal/mol for the 4FTA complex. Nevertheless, there is a low-energy minimum of the target function close to the native ligand pose for many of these complexes (INN = 1,2,…, 28,131). We can definitely conclude that the MMFF94* in vacuo* target function is invalid only for ligand positioning for four complexes (4FTA, 4FV6, 1DWC, and 1TOM): the energy of the optimized native ligand position is higher than energies of many target function local minima (IN ≫ 1) and there are no target function local minima close to the native pose (INN ≫ 1). Except for these four “bad” complexes, for all other complexes there is a local minimum of the target function (MMFF94* in vacuo*) which is situated near the ligand native pose and its index is not larger that ≈10^2^. This means that if we find 10^2^–10^3^ local minima of the target function (MMFF94* in vacuo*) there will be ligand poses among them which are situated near the native ligand pose or near the optimized native ligand pose. So, for the accurate calculations of the protein-ligand binding free energy we have to take into account the whole spectrum of the target function low-energy local minima. In this case, the local energy minimum (or several minima) near the native ligand pose or near the optimized native ligand position will be included in the calculation of the protein-ligand binding energy for many (for 13 complexes of 16) of the considered protein-ligand complexes.

We can see in Table 2, comparing “{1}MMFF94” and “{1}MMFF94 + PCM” columns, that taking solvent into account in the frame of the PCM method can improve the target function quality for most complexes, except for the 1VJ9 case. For example, the optimized native ligand position for the 4FV6 complex has too high energy (IN = inf) with the MMFF94* in vacuo* target function and all found by FLM low-energy minima are far from the native position (INN = inf). However, the energy recalculation for the same minima with the “{1}MMFF94 + PCM” target function put the optimized native ligand position into the global energy minimum (IN = 1). In other words, the “{1}MMFF94 + PCM” target function is more adequate for ligand positioning in 4FV6 complex than the “{1}MMFF94” target function does. Similarly for 4FV5 complex, transition from the “{1}MMFF94”* in vacuo* target function to “{1}MMFF94 + PCM” one results in the decrease of IN/INN indexes from 204/131 to 3/3.

Comparing the “{1}PM7” and “{1}PM7 + COSMO” columns, we can see similar target function quality improvement when the COSMO solvation model is taken into account. The semiempirical PM7 method* in vacuo* is not noticeably better than the MMFF94 force field* in vacuo* for ligand positioning: indexes IN/INN have similar values for both target functions (cf. “{1}PM7” and “{1}MMFF94” columns).

The second independent minima search with the FLM program (“{2}MMFF94”, “{2}MMFF94 + PCM”, and “{2}MMFF94 + SGB” columns) confirms the significance of taking into account solvent during minima selection and ranking; indexes IN/INN are noticeable lower for “{2}MMFF94 + PCM” and “{2}MMFF94 + SGB” target functions than for any other target functions presented in Table 2.

Also, comparing “{2}MMFF94 + PCM” and “{2}MMFF94 + SGB” columns in Table 2, we can conclude that the SGB method improves positioning quality better than the PCM method (e.g., IN/INN for 4FT0 decrease from 164/159 to 8/6 with changing the target function from “{2}MMFF94 + PCM” to “{2}MMFF94 + SGB”), although the PCM method is more sophisticated [28]. We explain this fact by relative smoothness of the SGB method comparing with PCM. The latter depends on the surface triangulation and the multicharge large matrixes approximation [35] which can be different for close local minima and demands many iterations to reach self-consistency. However, the SGB method is based on direct calculations and for close minima it must give close energies of protein-ligand complex interaction with solvent.

**Figure 2:**
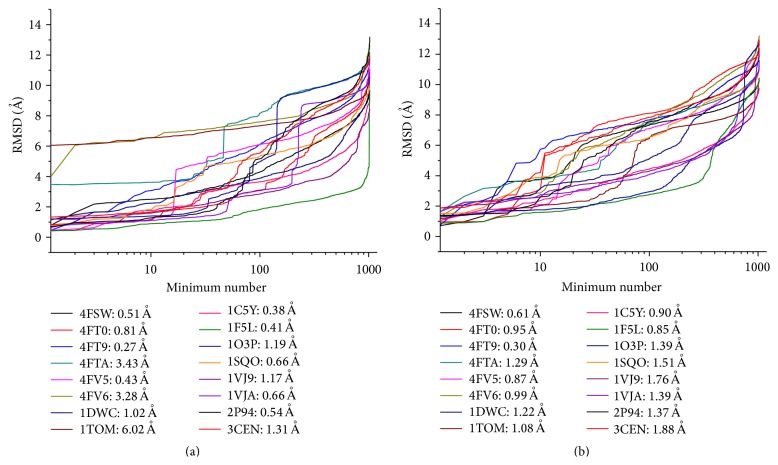
The distribution of the RMSD of the low-energy minima from the nonoptimized native ligand positions for 16 complexes for “{1}MMFF94” and “{1}MMFF94 + PCM” sets (a) and for “{2}MMFF94”, “{2}MMFF94 + PCM”, and “{2}MMFF94 + SGB” sets (b). The lowest RMSD values are presented for each complex in the insets.

Values of minima RMSD from the native (crystallized) ligand position are given in Figure 2 for all 1024 low-energy minima for each of all 16 complexes.  Figure 2(a) presents the RMSD of conformations obtained for the “{1}MMFF94” (and also for “{1}MMFF94 + PCM”) set, and Figure 2(b) presents the RMSD of conformations obtained for the “{2}MMFF94 + PCM” (and also for “{2}MMFF94” and “{2}MMFF94 + SGB”) set. Local optimization from the initial “{1}MMFF94” conformations carried out by the PM7 method and resulting in “{1}PM7” and “{1}PM7 + COSMO” sets did not change significantly the conformations of the ligands: RMSD between the initial “{1}MMFF94” and PM7 optimized poses were in the range ≈0.1–1.0 Å. All low-energy minima were ranked by the RMSD value and the relationships between the RMSD and order number are presented in Figure 2. It may be remarked that the lowest RMSD values are less than 2 Å for all complexes (excluding the 4FTA, 4FV6, and 1TOM complexes from the “{1}MMFF94” set).

The comparison of the RMSD values obtained by the FLM program with those obtained by other docking programs (SOL [16–18] and Autodock [8, 9]) shows that SOL docking with standard parameters (and 99 runs) gives 13 complexes out of 16 which have the smallest RMSD <2 Å, and Autodock docking with standard parameters (and 100 runs) gives 14 complexes out of 16 which have the smallest RMSD <2 Å. Thus, all these programs can find well enough the position near the native ligand position, but this position does not always correspond to the energy global minimum, which is defined by the energy target function.

**Figure 3:**
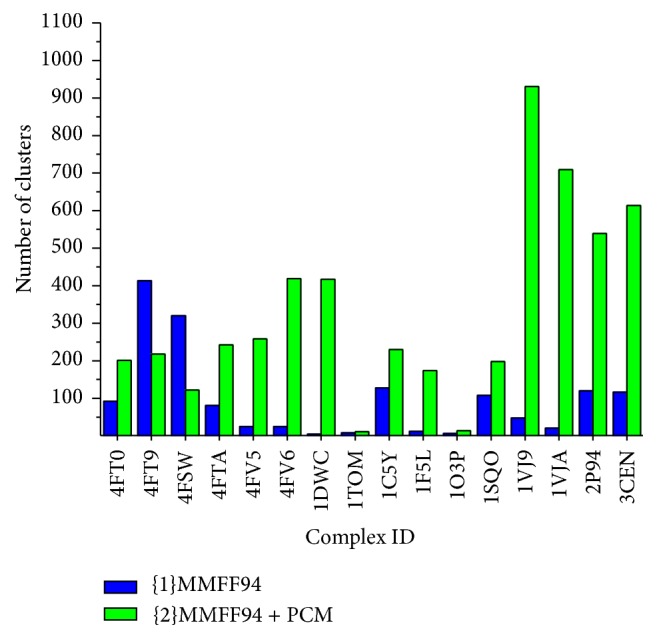
The number of clusters for each protein-ligand complex.

All low-energy minima found by FLM can be grouped into different clusters with respect to RMSD between the ligand conformations. Two conformations belong to one cluster if RMSD between them is less than 1.4 Å. Numbers of clusters for each protein-ligand complex are presented in Figure 3 and Table 3.

We can see that, for some complexes, 1TOM and 1O3P, all minima are divided into only several groups, but for many other complexes the number of clusters varies from several dozen to several hundred. Also, change from the energy in vacuum ({1}MMFF94) to the energy in solvent ({2}MMFF94 + PCM) during the minima search results in considerable increase in number of clusters for most of the complexes; that is, the diversity of low-energy minima configurations increases. Table 3 shows that the native ligand configuration does not always belong to the cluster with the lowest minima energies.

### 3.2. The Binding Free Energy Components

As the initial approximation for the binding energy, it is possible to take the difference between potential energy of the protein-ligand complex global minimum, potential energy of the free ligand global minimum, and potential energy of the free protein. These values for the MMFF94 force field in vacuum together with several additive corrections as well as the total binding energies (calculated in the multiwell approximation and experimental ones) are shown in Table 4.

As we can see from Table 4, the potential energy component of the binding energy is the most variable and important. The binding energy corrections, corresponding to translational and rotational degrees of freedom, are practically constant for all protein-ligand complexes, and binding energy corrections, corresponding to vibration degrees of freedom and multiple minima accounting, are relatively small. Thereby, the protein-ligand binding free energy is primarily determined by the potential energies of the global minima. We would like to emphasize that the energy of the ligand deformation from its free conformation to the bound one (the ligand strain energy) is automatically taken into account in our calculations, and its value is in the range from several kcal/mol to several dozen kcal/mol for tested complexes. This strain energy is neglected in the score of most docking programs.

### 3.3. Calculated and Experimental Binding Energies for Different Energy Functions

Finally, we compared experimental and calculated binding energies, and the results are presented in Table 5. For comparison of our results with those obtained through more conventional approaches and real docking programs, we also presented in Table 5 scoring functions obtained with docking programs SOL [16–18] and Autodock [8, 9]. The Autodock program is widely used for docking and the SOL program was successfully used recently for development of new thrombin [40], urokinase [41], and factor Xa inhibitors [42].

Experimental binding free energy Δ*G*_exp_ were obtained from the respective binding constants *K*_*i*_ by (5), where *K*_*i*_ are substituted in mol/L units and temperature *T* is equal to 310 K:

(5) $$\begin{array}{c} \Delta G_{exp}=kT*\ln\left(\;K_{i}\;\right). \end{array}$$

The “{1}MMFF94”, “{1}MMFF94 + PCM”, “{1}PM7”, and “{1}PM7 + COSMO” and “{2}MMFF94”, “{2}MMFF94 + PCM”, and “{2}MMFF94 + SGB” energies were calculated as a difference between potential energies of the protein-ligand complex (in the global minimum), free ligand (in the global minimum), and free protein (in its initial conformation, because it is considered as rigid).

The energy ranges (difference between the highest and the lowest energies among the 16 protein-ligand complexes) and coefficients of correlation with the experimental energies are also shown in Table 5. We can see that calculated binding energies differ strongly from the experimental values for all investigated energy functions (excluding the SOL and the Autodock scoring functions where the fitting coefficients are used). Nevertheless, we can see noticeable improvement in the values of the energy ranges when solvent is taken into account and quantum-chemical PM7 method is used instead of MMFF94 force field. Among seven variants of potential energy function, the “PM7 + COSMO” energy function has the most realistic energy range 35.5 kcal/mol to be compared with 7 kcal/mol for the experimental binding energy range.

Also we can see that the correlation coefficients are not close to 1, and for the “{1}MMFF94 + PCM”, “{2}MMFF94 + PCM”, and “{2}MMFF94 + SGB” cases the coefficient is even negative. But among seven variants of the potential energy function, the “PM7” energy function has the best correlation coefficient with experimental energies. We should emphasize that these results have been obtained by using the general purpose methods (MMFF94, PCM, PM7, and COSMO) without any fitting coefficients to the special case of the protein-ligand interactions.

Table 5 also presents the SOL and the Autodock scoring functions, their energy ranges, and their correlations with the experiment binding energies. Although the energy ranges of Autodock and SOL scoring functions are closer to the range of the experimental binding energies than the ranges of binding energies calculated by the FLM program with different target functions, the correlation coefficients of SOL and Autodock scoring functions with experimental values are much smaller than ones calculated with the FLM program (“{1}MMFF94”, “{1}PM7”, “{1}PM7 + COSMO”, and “{2}MMFF94”). This may follow from the fact that SOL and Autodock docking programs are aimed to virtual screening of large databases of compounds and many simplifications are used in these programs (e.g., simplified force field and absence of the ligand deformation energy in the scoring function).

## 4. Conclusions

The results of our investigations show that the docking target function on the base of MMFF94 force field* in vacuo* can be used for discovery of native or near native ligand positions for some protein-ligand complexes by finding the global energy minimum of the target function, for example, for five complexes of 16 in Table 2 either IN = 1 or INN = 1 (there were 14 such cases when we expanded the test set up to 30 complexes). If a set of low-energy minima of this target function is taken into account (not only the global minimum), we find the near native ligand position for much more complexes; for example, the number of such complexes in Table 2 will be 13 out of 16 for the set of 1024 low-energy minima. We can conclude that for the calculation of the protein-ligand binding free energy, it is better to take into account not only the global energy minimum but a whole set, for example, 1024, of the low-energy local minima. In this case, we take into consideration near native ligand positions for most of the complexes investigated in the present research. Nevertheless for some complexes (4FTA, 4FV6, and 1TOM in Table 2) it is impossible to find near native ligand positions among 1024 local minima of the target function (MMFF94* in vacuo*): indexes IN/INN are inf/inf. However, the rational target function improvement has been demonstrated without use of any fitting parameters: the ligand positioning is better for the target function of the more sophisticated physical model, that is, taking into account the implicit solvent model. Also improvement of the ligand positioning occurs when MMFF94 force field is substituted by the semiempirical quantum-chemical method PM7. It is apparent from Table 2 that indexes IN and INN decrease for the more sophisticated models. It is also obvious from Table 2 that usage of more sophisticated target function at the stage of low-energy minima selection results in better ligand positioning: the lowest IN and INN indexes are in the two rightmost columns in Table 2 and there are no “inf” labels at all in these two columns.

The best target functions of all target functions examined in this research are the protein-ligand potential energy in the frame of MMFF94 force field [22] with the implicit solvent SGB model [27, 28] and the potential energy in the frame of the semiempirical PM7 method [23] with the COSMO implicit solvent model.

The results of the present investigation show that further improvement of the docking target function is required until its global minimum coincides with or is near the optimized native ligand position for a broad set of protein-ligand complexes (IN = 1, INN = 1).

Calculated in the multiwell approximation binding free energies differ strongly from experimental binding energies for all investigated energy functions. More realistic binding energies were obtained for new quantum-chemical semiempirical PM7 method and taking into account water in the implicit COSMO model. The main contribution to the binding free energy is given by potential energies of the protein, ligand (in the global minimum), and their complex (in the global minimum) with solvent taken into account. Present investigations show that for the increase of docking accuracy for ligand positioning as well as for binding energy calculation it is necessary to take into account interaction of the protein, ligand, and their complex with water solvent and also to look for or create force field better than MMFF94 and/or to carry out docking with quantum-chemical methods, for example, with new semiempirical PM7 method.

The correlation coefficients between the experimental binding energies and the energies calculated by the FLM program are still far from unity, but for some target functions they are much larger than correlation coefficients between SOL or Autodock scoring functions and the experimental binding energies.

On the other hand, the improvement of the docking results for a given force field and a solvent model can be expected if we take into account the mobility of the protein atoms which locate close to the ligand (first of all, protein hydrogen atoms whose positions are not determined experimentally). Such a facility is included in the FLM program, and we hope to carry out respective investigations in the near future.

Despite the fact that parallel computing is used now mainly for docking of large ligand databases (virtual screening), the employment of advanced and more sophisticated models demands larger computational resources and increases importance of parallel algorithms and their acceleration [20] for docking of a single ligand.

Relying on the present investigations, we guess that current supercomputers are powerful enough to perform a comprehensive search of protein-ligand low-energy minima in the frame of existing two-body force fields with implicit solvent models and without usage of preliminary calculated energy grids for flexible ligands with up to about 20 internal torsions. The respective search programs (e.g., our program FLM) is a tool to evaluate adequacy of force fields and solvent models for ligand docking into the active sites of the target proteins.

## Supplementary Material

Supplementary materials present the structures and properties of the ligands from the analyzed complexes: PDB-identifier of the protein-ligand complex; total ligand charge in units of elementary charge; number of the ligand atoms including hydrogen; number of the ligand torsions (single acyclic bonds with rotating atoms); and 2D ligand chemical structures.

## Figures and Tables

**Table 1:** Parallelization efficiency of the FLM program: number of finished optimizations (*w*) in 3 hours depending on number of nodes (*N*), in other words, on number of working processes *n* = 8*∗N* − 1.

*N*	*n*	*w*	*w*/*n*
1	7	329	47
32	255	11567	45
1024	8191	379885	46

**Table 2:** IN/INN values for all tested 16 protein-ligand complexes and all 7 minima sets. “inf” for IN means that all (1024 minima) calculated low-energy minima have energy below the energy of the optimized native ligand. “inf” for INN means that all the low-energy minima found by FLM have RMSD from the native position above 2 Å.

PDB ID	{1}MMFF94	{1}MMFF94 + PCM	{1}PM7	{1}PM7 + COSMO	{2}MMFF94	{2}MMFF94 + PCM	{2}MMFF94 + SGB
4FT0	36/20	8/7	37/12	1/1	180/99	164/159	8/6
4FT9	45/28	1/1	25/6	1/1	194/125	3/1	1/1
4FSW	5/5	6/6	40/40	12/13	110/102	134/140	21/3
4FTA	inf/inf	4/inf	379/inf	1/inf	inf/inf	186/187	97/97
4FV5	204/131	3/3	253/194	2/1	186/134	6/3	5/5
4FV6	inf/inf	1/inf	49/inf	1/inf	86/289	3/68	1/24
1DWC	inf/670	245/25	689/661	158/141	245/114	250/35	107/8
1TOM	inf/inf	13/inf	inf/inf	1/inf	inf/inf	13/4	7/1
1C5Y	1/1	2/1	7/1	2/1	1/1	2/1	1/1
1F5L	1/1	1/1	43/16	69/30	1/1	10/1	1/1
1O3P	20/18	21/1	5/1	3/1	69/62	274/1	130/2
1SQO	1/1	2/1	1/1	1/1	1/1	54/1	5/1
1VJ9	46/1	86/51	32/1	26/8	6/1	11/18	10/14
1VJA	42/3	7/1	7/4	6/4	4/49	1/2	1/1
2P94	36/2	19/1	23/6	7/1	22/1	35/1	21/1
3CEN	96/1	18/1	13/1	3/1	90/1	35/1	13/1

**Table 3:** The indices of the clusters where the native ligand conformation is located. The clusters are sorted here by lowest energies of their conformations in ascending order; that is, the cluster containing the minimum with the lowest protein-ligand energy has index equal to 1. “inf” means that the native position does not fall into any cluster.

Complex ID	{1}MMFF94	{2}MMFF94 + PCM
	Cluster number with native pose	
4FT0	10	139
4FT9	29	1
4FSW	4	12
4FTA	inf	59
4FV5	10	3
4FV6	inf	23
1DWC	1	14
1TOM	inf	2
1C5Y	1	1
1F5L	1	1
1O3P	2	1
1SQO	1	1
1VJ9	1	inf
1VJA	2	2
2P94	2	1
3CEN	1	inf

**Table 4:** The protein-ligand binding energy components: potential energies and additive corrections to it. Δ*E* is the binding potential energy from the MMFF94 force field in vacuum, calculated by the global minima of the protein-ligand complex and the free ligand with energy of the free protein. Δ*G*_*ν*_ is the correction due to vibration degrees of freedom calculated with respective configuration integral *Z*_*ν*_ (2). Δ*G*_*t*_ and Δ*G*_*r*_ are corrections due to translational and rotational degrees of freedom calculated with configuration integrals *Z*_*t*_ (3) and *Z*_*r*_ (4), respectively. Corrections Δ*G*_*ν*_, Δ*G*_*t*_, and Δ*G*_*r*_ include both enthalpy and entropy components. Δ*G*_all_ is the correction for multiple minima accounting; it is calculated as a difference between binding free energy Δ*G*_bind_, calculated with multiple minima accounting, and binding free energy, calculated with only the global minima of the complex and the ligand. Δ*G*_exp⁡_ is the experimental binding energy calculated from the binding constant.

Protein	PDBID	Δ*G*_exp⁡_, kcal/mol	Δ*G*_bind_, kcal/mol	Δ*E*, kcal/mol	Δ*G*_*ν*_, kcal/mol	Δ*G*_*t*_, kcal/mol	Δ*G*_*r*_, kcal/mol	Δ*G*_all_, kcal/mol
CHK1	4FT0	−10.1	82.2	63.8	−3.9	10.6	10.3	1.4
	4FT9	−10.9	−48.5	−63.7	−5.0	10.4	9.9	−0.1
	4FSW	−6.8	−44.2	−60.2	−3.7	10.3	9.4	0.0
	4FTA	−9.8	−9.8	−30.4	−0.2	10.6	10.1	0.1

ERK2	4FV5	−10.9	−79.3	−102.1	0.5	10.7	10.6	1.0
	4FV6	−12.3	−74.6	−96.8	−0.4	10.8	10.7	1.1

Thrombin	1DWC	−10.5	−128.5	−144.9	−4.2	10.9	10.5	0.2
	1TOM	−11.8	−224.1	−248.7	2.7	10.7	10.5	0.7

Urokinase	1C5Y	−6.0	−16.3	−34.8	−0.7	10.0	8.7	0.5
	1F5L	−7.5	34.7	17.4	−2.3	10.2	9.3	0.1
	1O3P	−9.4	23.8	3.9	−2.0	10.6	10.2	1.1
	1SQO	−10.6	−4.6	−24.8	−0.4	10.3	9.6	0.7
	1VJ9	−10.7	−25.9	−50.7	2.4	11.0	10.8	0.6
	1VJA	−10.9	−31.0	−51.1	−2.1	10.9	10.5	0.8

Factor Xa	2P94	−13.0	−42.0	−68.2	−5.5	10.9	10.8	0.0
	3CEN	−11.7	−49.2	−69.5	−1.9	10.9	10.6	0.7

**Table 5:** Binding energies (in kcal/mol) calculated as *E*_0_^1^(PL)  −  *E*(P)  −  *E*_0_^1^(L), where *E*_0_^1^(PL) and *E*_0_^1^(L) are energies of the protein-ligand complex and the free ligand in their global minima, respectively, and *E*(P) is energy of the protein in its configuration prepared as it is described in Section 2. The global energies of complexes and ligands were taken from respective minima sets (see Section 2.2). Δ*G*_exp⁡_ is the experimental binding energy calculated from the binding constant. “Energy range” is the difference between the highest and the lowest energies among all 16 protein-ligand complexes. “Energy correlation” is Pearson correlation coefficient between experimental and calculated binding energies. Autodock and SOL scoring functions are also given to compare (in kcal/mol).

PDB ID	Δ*G*_exp⁡_	{1}MMFF94	{1}MMFF94 + PCM	{1}PM7	{1}PM7 + COSMO	{2}MMFF94	{2}MMFF94 + PCM	{2}MMFF94 + SGB	Sol Score	Autodock score
4FT0	−10.1	63.84	0.04	−39.48	−48.67	58.64	−0.08	3.03	−5.20	−7.15
4FT9	−10.9	−63.72	−9.98	−111.64	−48.77	−64.83	−10.05	−16.34	−4.29	−4.9
4FSW	−6.8	−60.20	−5.89	−108.48	−46.41	−60.19	−6.53	−7.72	−4.78	−6.08
4FTA	−9.8	−30.36	−5.44	−126.54	−58.61	−30.35	−15.04	−14.05	−4.35	−4.7
4FV5	−10.9	−102.01	−7.42	−168.44	−54.37	−102.01	−4.64	−11.16	−6.05	−8.25
4FV6	−12.3	−96.92	−7.75	−164.91	−72.68	−89.27	−13.42	−13.87	−5.26	−5.6
1DWC	−10.5	−146.16	−33.43	−194.69	−70.12	−144.88	−32.93	−36.77	−2.86	−4.24
1TOM	−11.8	−248.29	−49.66	−258.10	−73.67	−248.28	−49.89	−51.59	−8.11	−7.88
1C5Y	−6.0	−34.83	−79.15	−33.81	−52.84	−34.83	−79.34	−80.85	−6.83	−5.28
1F5L	−7.5	17.40	−52.26	−29.98	−81.95	17.40	−52.54	−52.88	−4.41	−6.62
1O3P	−9.4	3.59	−40.30	−32.53	−64.60	3.59	−40.59	−42.54	−6.95	−8.43
1SQO	−10.6	−24.78	−50.42	−58.75	−69.56	−24.78	−51.30	−52.22	−6.75	−8.68
1VJ9	−10.7	−49.57	−50.03	−91.69	−76.58	−49.20	−43.77	−49.18	−4.53	−3.17
1VJA	−10.9	−49.87	−51.91	−95.43	−81.40	−47.97	−38.86	−41.85	−4.47	−1.82
2P94	−13.0	−68.20	−15.74	−153.53	−68.94	−67.28	−15.79	−20.87	−6.53	−13.09
3CEN	−11.7	−69.48	−18.17	−133.10	−63.12	−69.47	−18.43	−26.62	−5.48	−11.68
Energy range	7.0	312	79.2	228	35.5	306.93	79.26	83.89	5.25	11.27
Energy correlation		0.41	−0.36	0.60	0.33	0.40	−0.39	−0.35	0.09	0.13

## References

[1] Mobley D. L., Dill K. A. (2009). Binding of small-molecule ligands to proteins: ‘what you see’ is not always ‘what you get’. *Structure*.

[2] Sadovnichii V. A., Sulimov V. B., Voevodin V. V., Sadovnichii V. A., Savin G. I. Supercomputing technologies in medicine. *Supercomputing Technologies in Science, Education, and Industry*.

[3] Merz K. M., Ringe D., Reynolds C. H. (2010). *Drug Design: Structure- and Ligand-Based Approaches*.

[4] Plewczynski D., Łaźniewski M., Augustyniak R., Ginalski K. (2011). Can we trust docking results? Evaluation of seven commonly used programs on PDBbind database. *Journal of Computational Chemistry*.

[5] Klimovich P. V., Shirts M. R., Mobley D. L. (2015). Guidelines for the analysis of free energy calculations. *Journal of Computer-Aided Molecular Design*.

[6] Chen W., Gilson M. K., Webb S. P., Potter M. J. (2010). Modeling protein-ligand binding by mining minima. *Journal of Chemical Theory and Computation*.

[7] Allen W. J., Balius T. E., Mukherjee S. (2015). DOCK 6: impact of new features and current docking performance. *Journal of Computational Chemistry*.

[8] Morris G. M., Goodsell D. S., Halliday R. S. (1998). Automated docking using a Lamarckian genetic algorithm and an empirical binding free energy function. *Journal of Computational Chemistry*.

[9] Huey R., Morris G. M., Olson A. J., Goodsell D. S. (2007). A semiempirical free energy force field with charge-based desolvation. *Journal of Computational Chemistry*.

[10] Rarey M., Kramer B., Lengauer T., Klebe G. (1996). A fast flexible docking method using an incremental construction algorithm. *Journal of Molecular Biology*.

[11] Claussen H., Buning C., Rarey M., Lengauer T. (2001). FLEXE: efficient molecular docking considering protein structure variations. *Journal of Molecular Biology*.

[12] Neves M. A. C., Totrov M., Abagyan R. (2012). Docking and scoring with ICM: the benchmarking results and strategies for improvement. *Journal of Computer-Aided Molecular Design*.

[13] Abagyan R., Totrov M., Kuznetsov D. (1994). ICM—a new method for protein modeling and design: applications to docking and structure prediction from the distorted native conformation. *Journal of Computational Chemistry*.

[14] Ewing T. J. A., Makino S., Skillman A. G., Kuntz I. D. (2001). DOCK 4.0: search strategies for automated molecular docking of flexible molecule databases. *Journal of Computer-Aided Molecular Design*.

[15] Cole J. C., Nissink J. W. M., Taylor R., Alvarez J., Shoichet B. (2005). Protein-ligand docking and virtual screening with GOLD. *Virtual Screening in Drug Discovery*.

[16] Sulimov A. V., Kutov D. C., Oferkin I. V., Katkova E. V., Sulimov V. B. (2013). Application of the docking program SOL for CSAR benchmark. *Journal of Chemical Information and Modeling*.

[17] Romanov A. N., Kondakova O. A., Grigoriev F. V. (2008). The SOL docking package for computer-aided drug design. *Numerical Methods and Programming*.

[18] Oferkin I. V., Sulimov A. V., Kondakova O. A., Sulimov V. B. (2011). Implementation of parallel computing for docking programs SOLGRID and SOL. *Numerical Methods and Programming*.

[19] Zheltkov D. A., Oferkin I. V., Katkova E. V., Sulimov A. V., Sulimov V. B., Tyrtyshnikov E. E. (2013). TTDock: a docking method based on tensor train decompositions. *Numerical Methods and Programming*.

[20] McIntosh-Smith S., Price J., Sessions R. B., Ibarra A. A. (2015). High performance *in silico* virtual drug screening on many-core processors. *International Journal of High Performance Computing Applications*.

[21] Cleves A. E., Jain A. N. (2015). Knowledge-guided docking: accurate prospective prediction of bound configurations of novel ligands using Surflex-Dock. *Journal of Computer-Aided Molecular Design*.

[22] Halgren T. A. (1996). Merck molecular force field. I. Basis, form, scope, parameterization, and performance of MMFF94. *Journal of Computational Chemistry*.

[23] Stewart J. J. P. (2013). Optimization of parameters for semiempirical methods VI: more modifications to the NDDO approximations and re-optimization of parameters. *Journal of Molecular Modeling*.

[24] Tomasi J., Persico M. (1994). Molecular interactions in solution: an overview of methods based on continuous distributions of the solvent. *Chemical Reviews*.

[25] Cramer C. J., Truhlar D. G. (1995). Continuum solvation models: classical and quantum mechanical implementations. *Reviews in Computational Chemistry*.

[26] Klamt A., Schüürmann G. (1993). COSMO: a new approach to dielectric screening in solvents with explicit expressions for the screening energy and its gradient. *Journal of the Chemical Society, Perkin Transactions*.

[27] Romanov A. N., Jabin S. N., Martynov Y. B., Sulimov A. V., Grigoriev F. V., Sulimov V. B. (2004). Surface generalized born method: a simple, fast and precise implicit solvent model beyond the Coulomb approximation. *Journal of Physical Chemistry A*.

[28] Kupervasser O. Y., Zhabin S. N., Martynov Y. B. (2011). Continual model of solvent: the DISOLV software—algorithms, implementation, and validation. *Numerical Methods and Programming*.

[29] FLM Program. http://dimonta2.1gb.ru/en/node/56.

[30] Byrd R. H., Lu P., Nocedal J., Zhu C. Y. (1995). A limited memory algorithm for bound constrained optimization. *SIAM Journal on Scientific Computing*.

[31] Zhu C., Byrd R. H., Lu P., Nocedal J. (1997). Algorithm 778: L-BFGS-B: fortran subroutines for large-scale bound-constrained optimization. *ACM Transactions on Mathematical Software*.

[32] http://hpc.msu.ru/.

[33] http://www.pdb.org/pdb/home/home.do.

[34] http://avogadro.openmolecules.net/wiki/Main_Page.

[35] Mikhalev A. Y., Oferkin I. V., Oseledets I. V., Sulimov A. V., Tyrtyshnikov E. E., Sulimov V. B. (2014). Application of the multicharge approximation for large dense matrices in the framework of the polarized continuum solvent model. *Numerical Methods and Programming*.

[36] http://openmopac.net/MOPAC2012.html.

[37] Mikhalev A. Y., Oseledets I. V. Adaptive Nested Cross Approximation of Non-Local Operators. http://arxiv.org/abs/1309.1773.

[38] Landau L. D., Lifshitz E. M. (1980). *Course of Theoretical Physics, Vol. 5, Statistical Physics*.

[39] Schwarzl S. M., Tschopp T. B., Smith J. C., Fischer S. (2002). Can the calculation of ligand binding free energies be improved with continuum solvent electrostatics and an ideal-gas entropy correction?. *Journal of Computational Chemistry*.

[40] Sinauridze E. I., Romanov A. N., Gribkova I. V. (2011). New synthetic thrombin inhibitors: molecular design and experimental verification. *PLoS ONE*.

[41] Sulimov V. B., Katkova E. V., Oferkin I. V. (2014). Application of molecular modeling to urokinase inhibitors development. *BioMed Research International*.

[42] Sulimov V. B., Gribkova I. V., Kochugaeva M. P. (2015). Application of molecular modeling to development of new factor Xa inhibitors. *BioMed Research International*.

