# Supplementary material for: Evaluation of Docking Target Functions by the Comprehensive Investigation of Protein-Ligand Energy Minima
Source: Adv Bioinformatics. 2015 Nov 26;2015:126858. doi: 10.1155/2015/126858 (PMC4674582; doi:10.1155/2015/126858)
Supplement: Supplementary file 1 — Supplementary materials present the structures and properties of the ligands from the analyzed complexes: PDB-identifier of the protein-ligand complex; total ligand charge in units of elementary charge; number of the ligand atoms including hydrogen; number of the ligand torsions (single acyclic bonds with rotating atoms); and 2D ligand chemical structures. [file 126858.f1.doc]

**Supplementary Material for manuscript «Evaluation of docking target functions by the comprehensive investigation of protein-ligand energy minima»**

*Igor V. Oferkin, Ekaterina V. Katkova, Alexey V. Sulimov, Danil C. Kutov, Sergey I. Sobolev, Vladimir V. Voevodin, Vladimir B. Sulimov*

**Table S1** The ligands structures and properties

| Protein | PDBID  and ligand  properties | Ligand chemical structure |
| --- | --- | --- |
| CHK1 | **4FT0**  Q=-1  N_A_=42  N_T_=4 |  |
|  | **4FT9**  Q=0  N_A_=32  N_T_=5 |  |
|  | **4FSW**  Q=0  N_A_=26  N_T_=0 |  |
|  | **4FTA**  Q=-1  N_A_=35  N_T_=6 |  |
| ERK2 | **4FV5**  Q=0  N_A_=52  N_T_=9 |  |
|  | **4FV6**  Q=0  N_A_=57  N_T_=14 |  |
| thrombin | **1DWC**  Q=0  N_A_=71  N_T_=14 |  |
|  | **1TOM**  Q=+2  N_A_=64  N_T_=11 |  |
| urokinase | **1C5Y**  Q=+1  N_A_=20  N_T_=2 |  |
|  | **1F5L**  Q=+1  N_A_=24  N_T_=6 |  |
|  | **1O3P**  Q=+1  N_A_=46  N_T_=6 |  |
|  | **1SQO**  Q=+1  N_A_=34  N_T_=4 |  |
|  | **1VJ9**  Q=+1  N_A_=74  N_T_=19 |  |
|  | **1VJA**  Q=+1  N_A_=61  N_T_=17 |  |
| factor Xa | **2P94**  Q=0  N_A_=60  N_T_=7 |  |
|  | **3CEN**  Q=0  N_A_=50  N_T_=7 |  |

The ligands from the analyzed complexes: PDB-identifier of the protein-ligand complex; total ligand charge in units of elementary charge, *Q*; number of the ligand atoms including hydrogen, *N_A_*; number of the ligand torsions (single acyclic bonds with rotating atoms), *N_T_*; and 2D ligand chemical structures.
